# Micro- and nanoplastics remodel the autophagy-lysosomal axis and mitochondrial function in primary human monocytes

**DOI:** 10.3389/fimmu.2026.1919477

**Published:** 2026-08-26

**Authors:** Stefania Pezzana, Martina Broggiato, Fiona Limanaqi, Elena Vezzoli, Silvia Zecchini, Luigia Valsecchi, Claudia Vanetti, Lara De Luca, Clara De Palma, Michela Bardini, Claudio Fenizia

**Affiliations:** 1 Department of Pathophysiology and Transplantation, University of Milan, Milan, Italy; 2 Department of Biomedical and Clinical Sciences, “L. Sacco” Hospital, University of Milan, Milan, Italy; 3 Advanced Light and Electron Microscopy BioImaging Centre (ALEMBIC), Università Vita-Salute San Raffaele, Milan, Italy; 4 Tettamanti Center, Fondazione Istituti di Ricovero e Cura a Carattere Scientifico (IRCCS) San Gerardo dei Tintori, Monza, Italy; 5 Department of Medical Biotechnology and Translational Medicine (BioMeTra), University of Milan, Milan, Italy; 6 Coordinated Research Center (CRC) Nano/microplastics on Environments, Medicine, Ecology, Systems Interaction Studies – NEMESIS, University of Milan, Milan, Italy

**Keywords:** human health, immune system, internalization pathway, microplastics, primary human monocytes, autophagy-lysosomal system, mitochondria, ultrastructure

## Abstract

**Background:**

Micro- and nanoplastics (MNPs) are increasingly detected in human tissues, raising concerns regarding their potential impact on immune cell function. Monocytes are among the first immune cells to encounter and internalize these particles. However, the intracellular mechanisms triggered by MNPs exposure remain poorly understood.

**Methods:**

Primary human monocytes were exposed to 25µg/mL of polystyrene (PS) MNPs ranging from 0.5 to 5 µm. Particle uptake and intracellular trafficking were investigated by transmission electron microscopy (TEM), while autophagy–lysosomal and mitochondrial responses were assessed by Western blotting, quantitative PCR, ultrastructural analyses, and metabolic flux measurements.

**Results:**

Ultrastructural TEM analysis revealed that primary human monocytes internalize MNPs through phagocytosis, followed by phagosome maturation and fusion with lysosomes. At a molecular level, MNPs exposure is associated with marked remodeling of the autophagy–lysosomal system, characterized by increased lysosomal abundance, elevated LC3-II/I ratio, and accumulation of autophagy-related vesicular structures. Although p62 protein levels increased following exposure, no corresponding changes were observed at the transcriptional level for key autophagy-associated genes, suggesting predominantly post-transcriptional regulation of these pathways. MNPs exposure also altered mitochondrial organization, increasing mitochondrial number while reducing mitochondrial area. Consistently, expression of the mitophagy-related gene *PINK1* increased, particularly following exposure to 1µm particles. Functional analyses demonstrated size-dependent mitochondrial dysfunction, with 0.5µm and 1µm particles reducing mitochondrial respiratory capacity, whereas 5µm particles exerted minimal effects.

**Conclusions:**

To our knowledge, this study provides the first integrated, size-resolved characterization of MNPs uptake and intracellular responses in primary human monocytes, combining ultrastructural and biological evidence of phagocytic internalization, autophagy-lysosomal flux, mitochondrial homeostasis, and cellular bioenergetics within the same experimental framework. MNPs uptake was associated with concurrent alterations in the autophagy–lysosomal axis, mitochondrial homeostasis, and cellular metabolism. Our findings further provide evidence that exposure to MNPs at 25µg/mL affects key intracellular pathways involved in immune cell homeostasis. These results highlight a potential link between particle accumulation and cellular alterations, which may have implications for myeloid cell function and long-term health.

## 1 Introduction

Plastic pollution represents a major and escalating global challenge, the full extent of which remains to be fully defined. Global plastic production has increased dramatically over recent decades, reaching nearly 413.8 million tons in 2023 (1), thereby contributing to a growing environmental burden worldwide. Through environmental weathering and mechanical fragmentation, larger plastic debris progressively breaks down into smaller fragments commonly classified microplastics (MPs, <5 mm) and nanoplastics (NPs, <1000 nm), which are highly persistent and tend to accumulate across ecosystems (2, 3). This fragmentation process affects multiple polymer types, including polystyrene (PS). PS is of particular relevance because it is widely used, environmentally persistent, and prone to fragmentation, contributing to the continuous release of micro- and nanoscale plastic (MNPs) particles into the environment (4, 5).

Emerging evidence indicates that MNPs particles can enter the human body through ingestion, inhalation, and dermal contact, and cross biological barriers to directly interact with cells. Indeed, MNPs have been detected in several human biological specimens including blood, lung tissue, placenta, and stool, raising growing concerns regarding their potential biological impact and their toxicological long-term consequences for human health (6–8). The clinical evidence of the presence of MNPs in several human organs highlight the broad and heterogeneous particle-size distribution ranging from 1 µm to 2000 µm (9–12). The innate immune system represents the first line of defense against foreign materials (13). Monocytes are precursors of macrophages and dendritic cells and are key actors in the gut, lung air-blood, and skin barriers. They play a central role in immune surveillance and are among the first immune cells to encounter, internalize, and clear foreign particles (13–16). Although previous studies showed MNPs accumulation into immortalized human and murine macrophages, little is known about the specific internalization process and the consequent downstream effects on primary human monocytes (17, 18).

Beyond their classical roles in phagocytosis and host defense, monocytes and macrophages contribute to tissue remodeling, clearance of apoptotic debris, and maintenance of tissue homeostasis, making them vulnerable to persistent intracellular stressors such as non-degradable particles (14, 15). Lysosomes are central components of the autophagy–lysosomal system, a highly conserved catabolic pathway responsible for the sequestration, delivery, and degradation of damaged organelles and other intracellular cargo. Accordingly, particle-associated alterations in lysosomal homeostasis may affect different stages of autophagic processing, including autophagosome–lysosome fusion and cargo degradation, potentially resulting in the accumulation of autophagic structures and altered turnover of cellular components (19). Furthermore, several studies have demonstrated that MNPs internalized by immune cells not only persist into lysosomal compartments, but also, they can trigger oxidative stress, cytokine production, and metabolic reprogramming, suggesting a link to mitochondrial function (20, 21). In fact, alterations of the autophagy-lysosomal pathway may trigger downstream mitochondrial dysfunction, ultimately compromising cellular metabolism (21).

Existing evidence indicates that MNPs exposure can perturb the autophagy–lysosomal pathway and promote persistent inflammatory activation (22–25). Therefore, elucidating the impact of MNPs on the autophagy–lysosomal system, mitochondria homeostasis and cellular metabolism in primary human monocytes is essential for defining the intracellular responses associated with particle accumulation and their potential long-term health implications.

Nevertheless, current studies mostly capture the overall cellular response to MNPs accumulation, without resolving the specific downstream molecular events that connect external particle exposure with internalization into primary human monocytes and the consequent impact on cellular functions (26). Also, it has been demonstrated that internalized MNPs can trigger oxidative stress, cytokine production, and metabolic reprogramming, suggesting a link to mitochondrial function (20, 21, 26). However, their intracellular fate after uptake including their persistence, trafficking, or possible clearance, remains poorly defined (25, 27, 28). Therefore, elucidating the intracellular processes altered by MNPs exposure in primary human monocytes is fundamental to better define the possible consequences of chronic particle exposure, which more closely reflects real-life environmental conditions.

Here, we performed an integrated, size-resolved characterization of the ultrastructural internalization and intracellular responses associated with polystyrene (PS) MNPs uptake in primary human monocytes. Specifically, we examined MNPs of different diameters at 25 µg/mL to assess particle internalization together with the associated autophagy–lysosomal, mitochondrial, and metabolic alterations under standardized experimental conditions.

Although environmental plastics are more heterogeneous in size, shape, and composition than the ones used in our experiments, and have been found across a wide range of concentrations in human tissues (10, 29, 30), standardized spherical PS spheres provide a controlled model to dissect how particle size influences intracellular pathways and metabolic responses in primary human monocytes under equal-mass exposure conditions (i.e. 25 μg/mL). Specifically, we examined particle uptake mechanism, lysosomal remodeling, autophagy dynamics, and mitochondrial function. We hypothesized that MNPs internalization engages the autophagy–lysosomal system and is associated with concurrent changes in mitochondrial function and cellular metabolism. Defining how MNPs perturb the autophagy–lysosome–mitochondria axis in primary human monocytes is essential to understand how chronic exposure to these contaminants may affect immune cell homeostasis and contribute to potential long-term consequences for human health.

## 2 Materials and methods

### 2.1 THP-1 cell culture and concentration

The human monocytic THP-1 cell line was cultured in suspension in RPMI (EuroClone, Milan, Italy) containing 4 mM L-glutamine supplemented with FBS (10%), 100 U/ml penicillin and 100 μg/ml streptomycin at 37 °C in 5% CO_2_. The sterility of the culture medium and reagents was maintained throughout the experiment through the meticulous adherence to aseptic protocols in a Class II biological safety cabinet. Furthermore, the cells were subjected to routine testing for mycoplasma contamination.

### 2.2 Isolation of peripheral blood mononuclear cells

Buffy coats were obtained through an established collaboration with Niguarda Hospital in Milan, Italy. The buffy coat was carefully aliquoted into 50 ml centrifuge tubes. The volume was brought up to 35 ml with phosphate-buffered saline (PBS, ECB4004L, EuroClone, Pero, MI, Italy). Peripheral blood mononuclear cells (PBMCs) were isolated via density gradient centrifugation using Ficoll (CL5020 Lympholyte-H, Cedarlane Laboratories Limited, Hornby, ON, Canada). 10 ml of Ficoll was gently added to the bottom of the 50 ml tube using a serological pipette and centrifuged at 2500 rpm for 25 minutes at room temperature (RT) without breaks. PBMCs were collected and washed with PBS. To remove remaining erythrocytes, 2 ml Erythrocyte Lysis Buffer (ECM0030D, Euro Clone, Pero, MI, Italy) was added, and the sample was incubated for 2 minutes at room temperature. The solution was then washed with PBS to stop the lytic activity and centrifuged for 8 minutes at RT at 1200 rpm. The supernatant was removed, and the pellet containing the PBMCs was resuspended in 10 ml of PBS to perform the cell count. All treatments and downstream assays were performed on freshly isolated monocytes.

### 2.3 Freshly isolated monocyte cell culture

Freshly isolated PBMCs were seeded in adherent 6-well plates (5-7 × 10^6^ cells/well), according to the experimental requirements, in RPMI medium (Euroclone, Milan, Italy) supplemented with 4 mM L-glutamine, 100 U/ml penicillin, and 100 μg/ml streptomycin. Cell density was adjusted based on an estimated monocyte amount of 10% within the PBMC population, to obtain the desired number of isolated monocytes. After 3 h of adhesion, non-adherent cells were removed, and adherent monocytes were maintained in the same medium further supplemented with 10% fetal bovine serum (FBS). This protocol was optimized in preliminary experiments, in which the purity of the isolated monocytes was assessed by flow cytometry and was consistently greater than 75%. Cell cultures were maintained at 37 °C in a humidified atmosphere containing 5% CO_2_. For autophagy-lysosomal-related studies, cells were treated with 100 nM Bafilomycin for 3h prior to cell harvest for Western blot analyses.

### 2.4 Treatment with MNPs

Yellow-green fluorescent (505/515) FluoSpheres™ Polystyrene Microspheres (Thermo Fisher Scientific) and non-fluorescent Polystyrene Microspheres (Thermo Fisher Scientific) with diameters of 0.1 μm, 0.5 μm, 1 μm, 5 μm, and 10 μm (**Supplementary Table 1**) were tested for endotoxin contamination with PYROGENT^®^ Plus Gel Clot LAL Assay (Cat. No. N294-125, Lonza) following manufacturer’s instructions and added to cell culture. To determine the optimal concentration to use, we first evaluated 0.1, 0.5, 1, 5, and 10 μm MNPs with concentrations ranging from 1 to 500 μg/mL on THP-1 cell line (n=1 per condition). All the experiments on primary human monocytes (n=3-6) were carried out using MNPs with diameters of 0.5 μm, 1 μm, 5 μm and a final concentration of 25 μg/mL. Exposure timepoints were selected according to biological process and experimental readout under investigation. For ultrastructural TEM imaging, and protein and gene quantification, primary human monocytes were exposed to MNPs for 24 and 72 hours to evaluate both early responses and later changes in autophagy-lysosomal system and mitochondrial rearrangement. Bioenergetic analyses were performed after 24 and 48 h of MNPs exposure to assess early and intermediate changes in mitochondrial respiration and glycolytic activity while limiting potential confounding effects associated with prolonged cell culture and reduced cell viability.

### 2.5 Flow cytometry

Flow cytometry was used to evaluate the effects of MNPs size and concentration on THP-1 cell recovery and particle internalization. THP-1 cells were exposed for 24 h to fluorescent polystyrene MNPs with diameters of 0.1, 0.5, 1, 5, or 10 µm at concentrations ranging from 5 to 5000 µg/mL. At the end of the exposure period, cells were collected in FACS tubes, centrifuged at 1200 rpm for 8 min at room temperature, and resuspended in PBS before acquisition. Samples were acquired using a CytoFLEX flow cytometer (Beckman Coulter, Pasadena, CA, USA). Cells were identified based on their forward-scatter and side-scatter properties, while debris, free MNPs, and particle-related events were excluded using a dedicated gating strategy. Live *vs* death cells were identified by Viakrome staining (Viakrome 405 Fixable Viability Dye, #C36614, Beckmann Coutler). MNPs internalization was assessed by determining the percentage of MNPs-positive cells and the mean fluorescence intensity (MFI) within the positive population. MFI was used as a relative index of the amount of fluorescent MNPs internalized by the cells. Untreated cells were used to define the negative population and establish the fluorescence threshold. Data were analyzed using Kaluza software (Beckman Coulter), and the same gating strategy (**Supplementary Figure 1**) was applied consistently to all samples.

### 2.6 MTT cell viability assay

Cell viability was assessed using the 3-(4,5-Dimethylthiazol-2-Yl)-2,5-Diphenyltetrazolium Bromide (MTT) assay. MTT reagent (Sigma-Aldrich) was dissolved in PBS at a final concentration of 5 mg/mL and sterilized by filtration. At the end of the treatment period, 30 µL of MTT solution was added to each well of a 96-well plate, followed by incubation for 4 h at 37 °C. The culture medium was then carefully removed, and the resulting formazan crystals were dissolved by adding 100 µL of DMSO to each well. The plate was protected from light with aluminum foil and placed on an orbital shaker for 15 min. Absorbance was measured at 570 nm using a Varioskan multimode plate reader within 60 min of formazan solubilization.

### 2.7 Transmission electron microscopy

To perform TEM imaging, the culture medium was removed and the cells were briefly washed once with 0.1 M sodium cacodylate buffer (pH 7.4, filtered) at room temperature (RT). Cells were fixed in 2.5% glutaraldehyde prepared in 0.1 M cacodylate buffer (pH 7.4) for 30 minutes at RT, followed by pelleting. They were then subjected to a secondary fixation at 4 °C for 24 h and post-fixed for 1 h in a reduced osmium solution containing 1.5% potassium ferricyanide and 1% aqueous osmium tetroxide in 0.1 M cacodylate buffer. After fixation, the pellets were washed with bi-distilled water at RT and incubated overnight at 4 °C in a 0.5% uranyl acetate solution. Dehydration was carried out using a graded ethanol series (70%, 80%, 90%, 100%) followed by acetone, with each step lasting 10 minutes. The samples were then embedded in Epon epoxy resin (Sigma, 45359) and cured at 60 °C for 48 hours. Non-serial ultrathin sections obtained from each cell pellet (for each donor and experimental condition) were obtained using a Leica UC7 ultramicrotome (Leica Microsystems, Vienna, Austria), mounted onto 300-mesh copper grids, and imaged with a Talos L120C G2 transmission electron microscope (Thermo Fisher Scientific Inc., Waltham, MA, USA) at an acceleration voltage of 120 kV. Images acquired at up to 8,500× magnification were used to quantify MNPs number, mitochondrial number, mitochondrial size, and vacuole number per cell profile by two independent experienced investigators blinded to the experimental condition. For each donor and experimental condition, multiple non-serial ultrathin sections distributed across several grids were systematically examined until at least 20 complete cell profiles had been acquired. To ensure representative sampling and minimize sampling bias, grid squares were scanned from one corner using regularly spaced parallel sweeps across the sectioned cell pellets. Cell profiles encountered during this systematic scanning procedure were acquired without phenotype-based selection.

For each complete cell profile, MNPs, as well as mitochondria, and vacuoles were counted according to specific predefined morphological criteria that were applied consistently to all experimental groups. Mitochondria were counted only when the outer membrane was clearly distinguishable, and the organelle could be unequivocally identified based on characteristic double membrane and cristae. Mitochondrial circularity was calculated according to the formula circularity = 4π (area/perimeter²). Vacuoles were identified as membranous vesicular compartments displaying clear or variably electron-dense lumen, with or without heterogeneous intracellular content, including endocytic, autophagic or lysosomal-like structures. Only membrane-delimited vacuolar structures entirely contained within the cell profile were included in the quantification. Organelle abundance was quantified as the percentage of the total cytoplasmic area occupied by the organelle of interest. For each cell profile, the organelle area was manually delineated on TEM images and divided by the corresponding total cytoplasmic area, excluding the nucleus. The result was expressed as % organelle area = (total organelle area/total cytoplasmic area) × 100. Finally, MNPs were readily distinguished from endogenous intracellular structures by their spherical morphology, smooth and sharply demarcated contour, homogeneous electron-lucent interior, apparent diameter compatible with sectioned polymeric MNPs, and the lack of any internal ultrastructural organization.

All the analyses were performed with ImageJ software (version 1.47, NIH). Organelle and MNPs counts were first expressed as the number of organelles or MNPs per cell. Cell-level values were then averaged to obtain a single mean value for each donor and experimental condition. Statistical analyses were performed using donor means as independent biological replicates, and data were expressed as mean **±** SEM.

Although measurements derived from ultrathin two-dimensional sections are affected by section-plane sampling and do not provide absolute three-dimensional estimates of organelle number or volume, all samples were processed, embedded, sectioned, imaged and quantified using identical procedures. Thus, quantitative measurements expressed per cell (i.e., per complete cell profile) provide reliable and internally consistent relative comparisons between experimental groups.

### 2.8 Microscopy and immunofluorescence

PBMCs were seeded on coverslips placed in a 24-well plate, which had been previously treated with fibronectin 1-5µg/cm^2^ to facilitate adhesion of monocytes. The cells were then incubated with as previously described. At 24 and 72 hours, the cells were fixed in PBS 4% paraformaldehyde (PFA) at RT for 10 minutes and were then permeabilized with 0.1% Triton X-100 in PBS for 10 minutes. Nuclei were stained using PureBlu™ DAPI (Biorad) at a concentration of 1:1000 for 30 minutes at RT. LC3B was marked using primary rabbit anti-LC3B antibody (1:1000, Cell Signaling, #43566S) and the respective anti-rabbit (1:5000, Cell Signaling, #7074S) secondary antibody. For LysoTracker-based lysosomal staining, 30 min prior to fixation, cells were incubated with 50 nM LysoTracker^®^ Red DND-99 (Cat L7528, Thermo-Fisher Scientific). Finally, confocal microscope images at a resolution of 1024 × 1024 pixels were acquired with a TCS SP8 system, equipped with a DMi8 inverted microscope and an HC PL APO 40×/1.30 Oil CS2 (Leica Microsystems, Wetzlar, Germany).

### 2.9 RNA extraction from primary monocytes

For transcriptional analyses, cells were resuspended/collected in RNAzol^®^ (TEL-TEST Inc., Friendswood, TX, United States). Chloroform (Sigma-Aldrich, C2432-1L) was added to the sample at a ratio of 1:5. The microtubes were shaken vigorously for 3 minutes at RT in order to obtain a homogeneous sample. Then, samples were centrifuged at 13000 rpm for 15 minutes at 4°C. At the end of the centrifuge, the aqueous phase containing RNA was transferred in a new tube where isopropanol (#I9516, SIGMA-ALDRICH Sigma-Aldrich) was then added in a 1:1 proportion. Microtubes were then vortexed and incubated overnight at -20 °C to allow RNA precipitation. At the end of the incubation, the sample was gradually thawed at 4°C for 15 minutes, then centrifuged at 13000 rpm for 15 minutes at 4°C. After centrifugation, the supernatant was discarded, and the RNA pellet was washed twice with 200 μl of 75% ethanol (#1.00983.2511, Sigma-Aldrich) by centrifugation at 13000 rpm for 15 minutes at 4°C. The pellet was then left to dry and then was finally resuspended in 11 μl of nuclease-free water to proceed with quantification by the Nanodrop 2000 Instrument (ThermoScientific, Waltham, MA, United States).

### 2.10 RNA reverse transcription to cDNA

For each sample, 1 μg of RNA was reverse transcribed using the following mixtures. To remove DNA contamination, a DNase mix was added to each sample, consisting of 0.25 μl of DNase I enzyme (M0303S, New England BioLabs) and 1.2 μl of 10× DNase buffer (B0303S, New England BioLabs), for a total volume of 1.45 μl. The reaction was then incubated at 37°C for 10 minutes to activate DNase. To inhibit DNase activity, 1 μl of 60mM EDTA was added and samples were incubated at 75°C for 10 minutes. Then, 1 μl of Oligo-random primer mix was added to each microtube. This mix consisted of nuclease-free H_2_O (60%), Oligo(dT) primers (20%; #C110A, 500 μg/ml, Promega) and random primers (20%; #C1181, 500 μg/ml, Promega). The tubes were then placed back in the thermoblock at 70°C for 5 minutes. The samples were placed on ice immediately after the 5-minute period. Each tube then received 6.5 μl of RT enzyme reaction mix, consisting of 4 μl of 5× RT buffer (#M531A, Promega), 1 μl of RT enzyme (200 U/μl; #M1708, Promega), 1 μl of dNTP mix (dTTP U123A, dGTP U121A, dCTP U122A, dATP U120A; 100 mM, Promega), and 0.5 μl of RNase inhibitor (RNasin, 40 U/μl; #N2113, Promega). The reaction was incubated at 42°C for 60 minutes, followed by 95°C for 5 minutes to inactivate the enzyme. The resulting cDNA samples were then stored at −20 °C.

### 2.11 Real time quantitative PCR

cDNA (25 ng) was amplified and quantified by real-time qPCR (CFX96 connect, Bio-Rad, Hercules, CA, United States) through the Universal SYBR^®^ Green Supermix (Bio-Rad, Hercules, CA, United States) in a final reaction mix volume of 10 μL. PrimePCR™ PreAmp for SYBR^®^ Green Assay for LAMP1, LC3, p62/SQSTM1, FIS1, PINK1, NRF1, BAX, OPA1, ULK1 MT-ND1, TOMM20, and TFAM genes were purchased from Bio-Rad (Bio-Rad, Hercules, CA, United States). Primer for GAPDH (F: 5’-CGGATTTGGTCGTATTGGG-3’; R: 5’-GCTTCCCGTTCTCAGCCTTG-3’) was purchased from Sigma-Aldrich. cDNA was amplified using the following thermal cycle: one hold at 95°C for 15 minutes, followed by 40-cycles at 5°C for 15 seconds, 60°C for 1 minute, and 72°C for 20 seconds. Each analysis included negative controls. A threshold cycle value (Ct) of 35 or higher was considered non-significant. The fusion curves were also analyzed to exclude artefacts. Dedicated software (CFX-Maestro) allows automatic background subtraction by returning normalized Ct values. Gene expression results were calculated based on the 2-ΔΔCt equation and presented as the average of the relative percentage of normalized expression relative to the gene housekeeping GAPDH.

### 2.12 Western blot analysis

Cells were lysed in ice-cold RIPA lysis buffer supplemented with 2% SDS, and a cocktail of protease and phosphatase inhibitors (cOmplete and PhosSTOP; Roche Applied Science, Mannheim, Germany), sonicated, incubated on ice for 30 min on a platform rotator, and then centrifuged at maximum speed for 30 min at 4°C. Protein concentration from whole cell lysates was determined through BCA protein assay kit (Pierce, United States). 30-to-40 μg proteins per sample were prepared by combining the appropriate volume with 4 ×Reducing Laemmli SDS sample buffer (final 1×, J60015.AC, Thermo-Fisher Scientific) and Milli-Q H2O. Samples were boiled for 5 min and loaded on 4%-12% Mini-Protean Stain-Free TGX Precast SDS-PAGE gel (4,568,095, Bio-rad, Hercules, CA, United States), which was run at 100 V for 1.5 h. The gel was activated at ChemiDoc MP imaging system (Bio-Rad, Hercules, CA, United States) and total proteins were transferred on PVDF membrane through the Trans-Blot Turbo Transfer System TM and Transfer Pack TM (Bio-Rad, Hercules, CA, United States). The stain-free membrane was imaged at ChemiDoc MP imaging system (Bio-Rad, Hercules, CA, United States) and then blocked in 1×TBS with 5% Bovine Serum Albumin (BSA) for 1 h at RT, washed in TBS-0.1% Tween (TBS-T), and incubated overnight at 4°C with the following antibodies prepared in 2.5% BSA: rabbit anti-LC3B (1:1000, Cell Signaling, #3868), mouse anti-SQSTM1/p62 (1:1000, cell signaling, #88588), mouse anti-LAMP1 (1:1000, cell signaling, #15665). The day after, the blot was washed 3 × 5 min with 1×TBS-T and incubated for 1 h with HRP-conjugated secondary antibodies goat anti-rabbit (1:5000, cell signaling #7074S) or goat anti-mouse (1:5000, cell signaling #7076S) in blocking buffer with 2.5% BSA. The blot was washed 3 × 5 min with 1×TBS-T and then incubated for 5 min with Clarity Western ECL substrate and visualized with a ChemiDoc MP imaging system (Bio-Rad, Hercules, CA, United States). For membrane reprobing, a Renew stripping buffer (SBS069,0500, Cyanagen, Bologna, Italy) was used for approximately 20 min under constant agitation, followed by 3 × 5 min washes in 1×TBS-T and 1 h incubation in blocking solution. Results were analyzed using the Image Lab software (Bio-Rad, Hercules, CA, United States). Quantification was performed through normalization to total protein. For each experiment, a representative blot is shown.

### 2.13 Seahorse bioenergetic profiling

Mitochondrial respiration in primary human monocytes was assessed using the Seahorse XF Cell Mito Stress Test kit (Agilent technologies, #103015-100, CA, United States) on a Seahorse XF Pro Analyzer (Agilent Technologies). Primary monocytes were isolated from peripheral blood of healthy donors (LS Columns, # 130-042-401, Miltenyi Biotec) and seeded in Seahorse XF cell culture microplates at the concentration of 60’000 cells/well on seahorse 96-well plates were pre-coated with poly-D-lysine (Sigma-Aldrich). After seeding, cells were exposed to 0.5, 1 and 5 µm PS-MNPs for 24 or 48 h.

At the end of the exposure period, cell culture media was replaced with Seahorse XF assay media (XF RPMI, pH 7.4) supplemented with 10 mM glucose, 2 mM glutamine, and 1 mM pyruvate. Cells were then incubated for 60 min at 37 °C in absence of CO_2_ to allow temperature and pH equilibration. Oxygen Consumption Rate (OCR), an indicator of mitochondrial activity, and Extracellular Acidification Rate (ECAR), a marker of glycolytic activity, was measured real-time in basal conditions and after sequential injection of Oligomycin (1.5 μM), BAM15 (2.5 μM) and Rotenone/Antimycin (0.5 μM). Compound concentrations were previously optimized through titration experiments. OCR, ECAR, total ATP production (MitoATP) and functional parameters of mitochondrial respiration (basal respiration, maximal respiration and spare respiratory capacity) were automatically calculated by Seahorse Wave Pro software and Seahorse Analytics (Agilent). Each value was calculated as the mean of six to eight technical replicates and normalized to the total number of nuclei per well. For normalization, the nuclei were stained with Hoechst-33342 dye and images were acquired by Operetta CLS to for nuclei count.

### 2.14 Statistical analysis

The GraphPad Prism software package (GraphPad Software, San Diego, CA, United States) was used to generate all the graphs and perform statistical analyses. The statistical significance was tested through one-way, or two-way ANOVA, followed by Fisher’s least significant difference (LSD) *post-hoc* test, or Tukey’s multiple-comparison correction. In detail, Fisher’s LSD test was applied to analyses based on pre-specified, hypothesis-driven comparisons, in which the biologically relevant comparisons were defined *a priori* (primarily each treatment versus its corresponding untreated control and, where appropriate, the same treatment between exposure times). In these analyses, unrestricted pairwise comparisons among all particle sizes were not the objective, and Fisher’s LSD was therefore appropriate following a significant ANOVA. Conversely, for analyses in which comparisons among all particle sizes were biologically relevant, we applied Tukey’s multiple-comparison correction. Individual p values were shown on the graphs. Results are reported as mean ± SEM of independent biological replicates (donors, n ≥ 3). Exploratory Spearman correlation analysis was performed using paired mRNA and protein data about autophagy and mitochondrial-related markers obtained across control and MNP-treated conditions (n=24 paired observations from n=3 independent donors). Statistical significance was set at p ≤ 0.05. All procedures were performed in accordance with the GLP guidelines adopted in our laboratories.

## 3 Results

### 3.1 Optimization and validation of MNPs exposure conditions in THP-1 cell line and primary human monocytes

**Figure 1:**
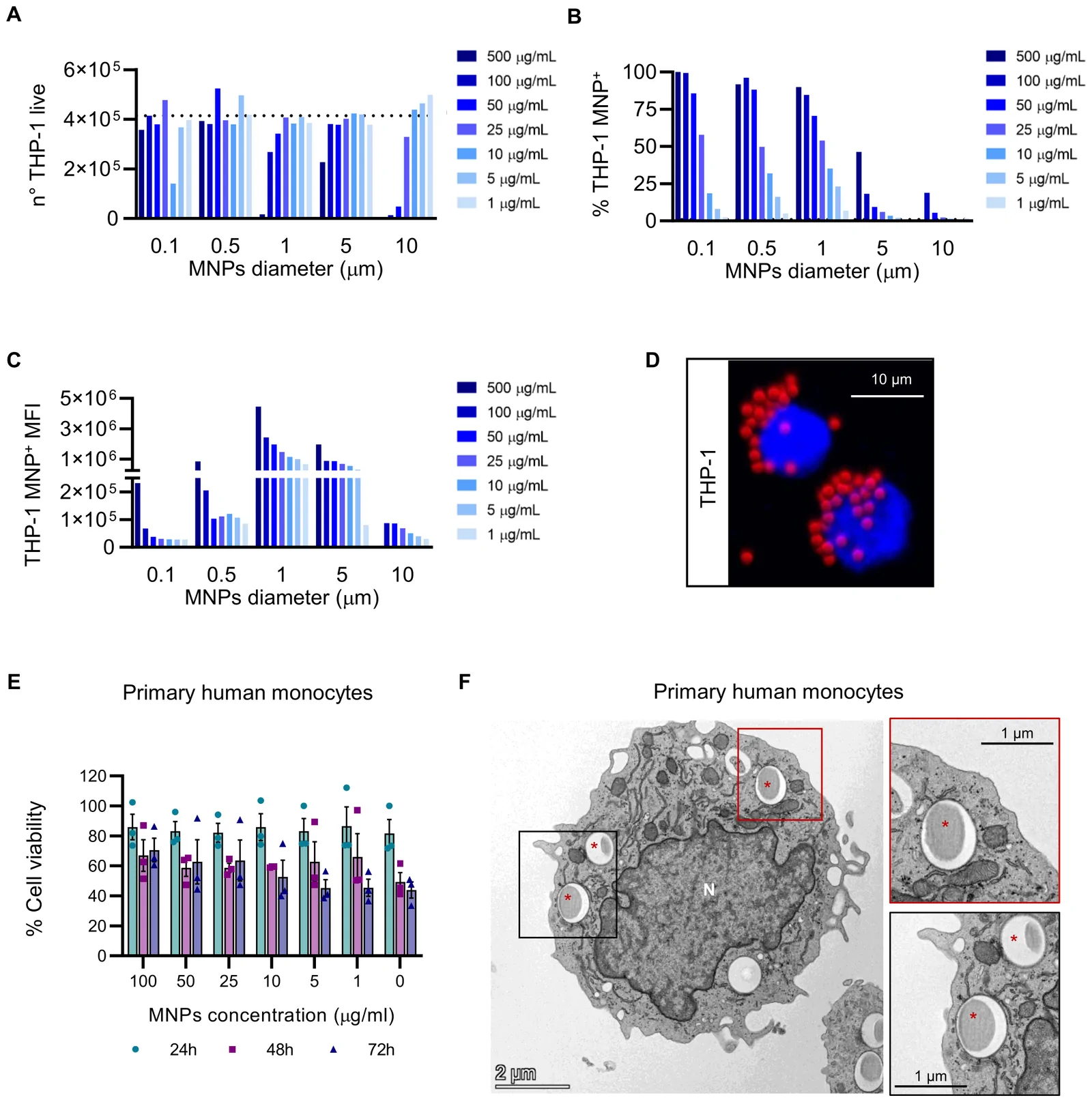
Size- and dose-dependent effects of MNPs. **(A)** Live THP-1 cell counts, **(B)** % of THP-1 cell MNP^+^, and **(C)** mean fluorescence intensity (MFI) of MNP^+^ cells after 24 h exposure to fluorescently labelled MNPs with diameter of 0.1, 0.5, 1, 5, 10 µm at concentrations ranging from 1 to 500 µg/ml. **(D)** Representative confocal images of THP-1 cells after 24 h exposure to 1 µm diameter MNPs with at 25 µg/ml. *Red: MNPs; Blue: nuclei*. **(E)** Viability of freshly isolated human monocytes at 24, 48, and 72 h post exposure to 1 µm MNPs at ranging from 1 to 100 µg/ml. **(F)** Representative TEM images of primary human monocytes after 24 h exposure to 1 µm MNPs (25 µg/mL). Red asterisks indicate MPs; N, nucleus. Pilot THP-1 study n=1; primary monocytes experiments n= 3 biological replicates/condition. Data are mean ± SEM. Two-way ANOVA, followed by Fisher’s LSD *post-hoc* test performed at donor-level means (n=3).

We first performed a pilot study in the immortalized human monocyte cell line THP-1 to define a MNPs concentration–size window that yields measurable particle uptake while preserving viability over time. THP-1 cells were exposed to fluorescent polystyrene (PS) MNPs ranging from 0.1–10 µm at concentrations of 1–500 µg/mL. Flow cytometry was used to quantify cell viability (**Figure 1A**) and particle uptake (**Figures 1B, C**). Cell viability was largely preserved across all tested conditions, with a reduction observed only at the highest concentration (500 µg/mL) for 1–10 µm MNPs, whereas 10 µm MPs showed a low cell viability at 100-25 µg/mL (**Figure 1A**). Accordingly, concentrations ≤100 µg/mL were considered viability-sparing. Among MNPs sizes, 0.1 µm particles showed the most heterogeneous viability across doses (**Figure 1A**). In contrast, MNPs particle association (percentage of MNP^+^ cells) increased in a concentration-dependent manner and plateaued at higher doses for MNPs between 0.1 and 1 µm (**Figure 1B**). Uptake was also size-dependent, with 0.1–1 µm particles showing higher association compared to larger particles (≥5 µm) at comparable concentrations, consistent with more efficient internalization. Finally, mean fluorescence intensity (MFI) of MNP^+^ cells increased with concentration, with 1 µm producing the strongest detectable signals, followed by 5 µm MNPs (**Figure 1C**). Lower signals for 0.1–0.5 µm particles were consistent with instrument detection limits, whereas the weak signal observed for 10 µm particles was consistent with their limited cellular uptake (**Figure 1C**). Representative confocal images of THP-1 cells exposed for 24 h to 1 µm MNPs support their intracellular localization (**Figure 1D**).

To validate these findings in a physiologically relevant primary cell system, freshly isolated human monocytes from three donors were exposed to 1 µm MNPs (1–100 µg/mL), and cell viability was assessed by MTT assay at 24, 48, and 72 h. Viability remained ~80% at 24 h across all concentrations and declined to ~60–70% at 48 and 72 h in both MNPs-treated cells and untreated controls. Although this time-dependent reduction suggests increasing baseline stress under prolonged culture conditions, typical of unstimulated primary cells, no additional dose-dependent decrease was observed in response to MNPs exposure within the concentration range tested (**Figure 1E**). Transmission electron microscopy (TEM) confirmed intracellular uptake and cytoplasmic localization of 1 µm MNPs at 24 h post-exposure (**Figure 1F**).

Based on these results, we selected 0.5, 1, and 5 µm particles at 25 µg/mL (within a 10–100 µg/mL working range) and exposure times of 24–72 h for subsequent mechanistic studies in primary monocytes, as these conditions preserved viability and yielded robust uptake.

### 3.2 Ultrastructural analysis of MNPs internalization and intracellular fate in primary monocytes

**Figure 2:**
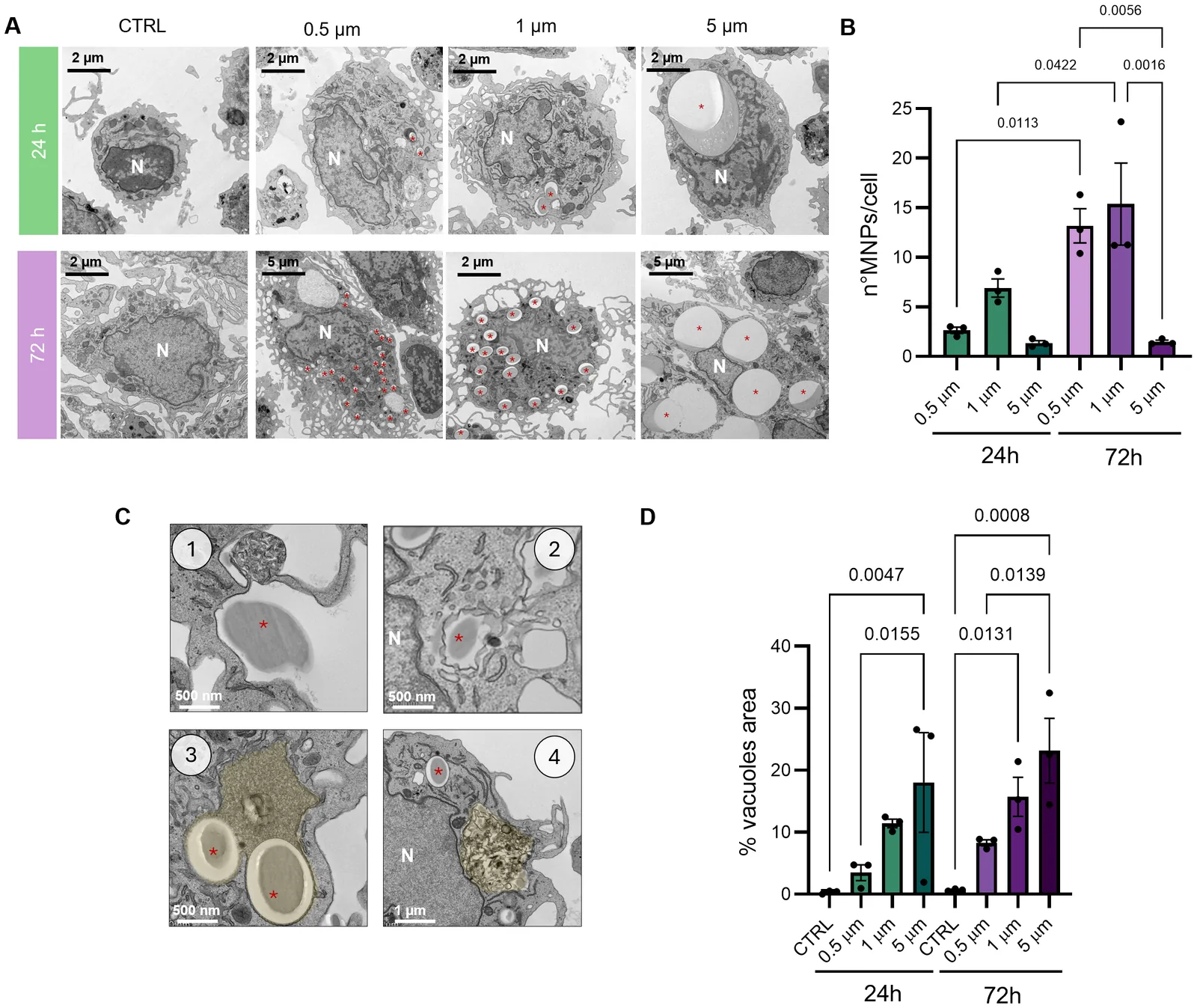
Ultrastructural analysis of MNPs uptake and intracellular fate in primary human monocytes. **(A)** Representative TEM images of freshly isolated monocytes at 24 and 72 hours post exposure to 25 µg/ml of MNPs with diameter of 0.5, 1, and 5 µm. **(B)** Quantification of the number of MNPs internalized per cell. **(C)** Representative TEM images illustrating sequential stages of MNP uptake and processing: (1) membrane engagement and phagocytic cup formation; (2) early phagosome formation; (3–4) phagosome maturation and phagolysosome formation, characterized by the presence of electron-dense degradative organelles (highlighted in orange) and increasing intraluminal density consistent with lysosomal degradation. **(D)** Quantification of degradative organelles area per cell under the indicated conditions calculated as % vacuoles area = area of degradative organelles (µm^2^)/total cytoplasm area (µm^2^). Data show size- and time-dependent increases in intracellular vesicular compartments. n= 3 biological replicates/condition. Data are mean ± SEM. Two-way ANOVA, followed by **(B)** Tukey and **(D)** Fisher’s LSD *post-hoc* tests. Red asterisks indicate MNPs; N: nucleus.

To define how freshly isolated human monocytes internalize and process MNPs, we performed longitudinal ultrastructural analyses by TEM imaging. We first quantified the single-cell particle burden following exposure to 0.5, 1, and 5 µm MNPs (25 µg/mL) for 24 and 72 h (**Figure 2A**). For 0.5 and 1 µm, the mean number of particles per cell increased with exposure time, indicating progressive accumulation. Accordingly, particle burden was higher at 72 h compared to 24 h (**Figure 2B**). Although a higher uptake trend was observed for 1 μm compared with 0.5 μm MPNs, this difference was not statistically significant.

In contrast, 5 µm MNPs showed limited intracellular presence, with no appreciable increase over time (**Figures 2A, B**), consistent with flow cytometry measurements of reduced particle association (**Figure 1B**). We next examined ultrastructural features of MNPs uptake (**Figure 2C**), revealing a sequence of morphological, membrane-associated internalization events consistent with phagocytosis, namely (i) membrane engagement and phagocytic cup formation, characterized by plasma membrane ruffling and pseudopod extension around MNPs, with continuity between the forming cup and the extracellular space; (ii) phagocytic cup closure and early phagosome formation, in which particles are enclosed within a single membrane-bound compartment containing an electron-lucent lumen; and (iii–iv) phagosome maturation and phagolysosome formation, characterized by larger single-membrane vesicles containing multiple particles and progressively electron-dense intraluminal material, consistent with degradative processing (31).

**Figure 3:**
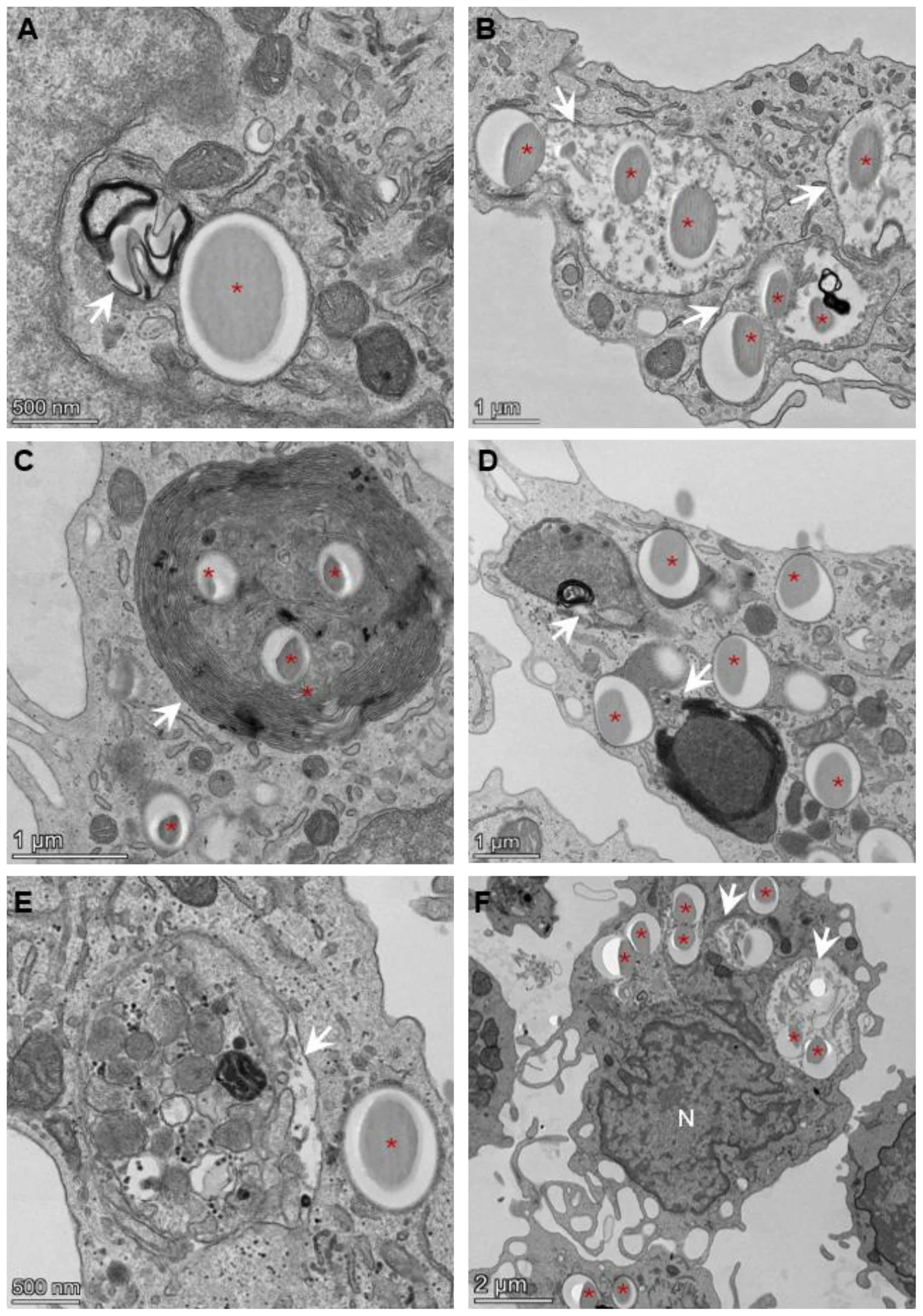
Ultrastructural features of MNP-containing vacuoles and associated remodeling of degradative vacuolar hubs with autophagic features. **(A–F)** Representative TEM images of human monocytes exposed to MNPs at 24 or 72 h. **(A)** MNP-containing phagosome surrounded by multilamellar membrane structures (lamellar whorls; arrow). **(B)** Multiple intracellular MNP-containing vacuoles (arrows), displaying heterogeneous morphology and electron density. **(C)** Prominent multilamellar body (arrow) adjacent to MNP-containing compartments. **(D)** Coexistence of MNP-containing phagosomes (arrows) with vacuolar structures of variable size and content, including early and late phagolysosomal compartments. **(E)** Giant degradative vacuolar compartment (arrow) resembling an autolysosome/phagolysosome, containing heterogeneous material including organelles and electron-dense debris. **(F)** Overview of a cell displaying extensive accumulation of MNP-containing vacuoles (arrows) and enlarged vacuolar compartments. Red asterisks indicate MNPs; N, nuclei.

In line with the appearance of phagocytic and degradative-like vesicular structures across all MNPs sizes examined, the total vesicular area per cell increased with both particle size and exposure time, supporting size- and time-dependent uptake and intracellular sequestration (**Figure 2D**). Beyond features consistent with phagosomes and phagolysosomes, we observed prominent multilamellar bodies (lamellar whorls) in proximity to, or even encasing, MNP-positive vacuoles (**Figures 3A–F**). Such structures are commonly associated with endo-lysosomal remodeling under conditions of increased autophagic–lysosomal membrane turnover, including elevated intracellular cargo load. Here, adjacent to the MNP-containing phagosomes, large vacuolar compartments reminiscent of degradative hubs could also be detected. These compartments contained mitochondria, multivesicular bodies, and secondary lysosomes/phagolysosomes with heterogeneous electron density, some with intraluminal vesicles, and degradative debris. These features are compatible with active degradation typical of the autolysosome/phagolysosome stage (**Figures 3A–F**). Taken together, these ultrastructural features support phagocytosis as the primary entry route for 0.5–5 µm MNPs and suggest that, following internalization, MNPs engage the degradative vacuolar system. However, their persistence appears to impose a substantial burden on this pathway, potentially perturbing endo-lysosomal homeostasis and implicating autophagy-related processes.

**Figure 4:**
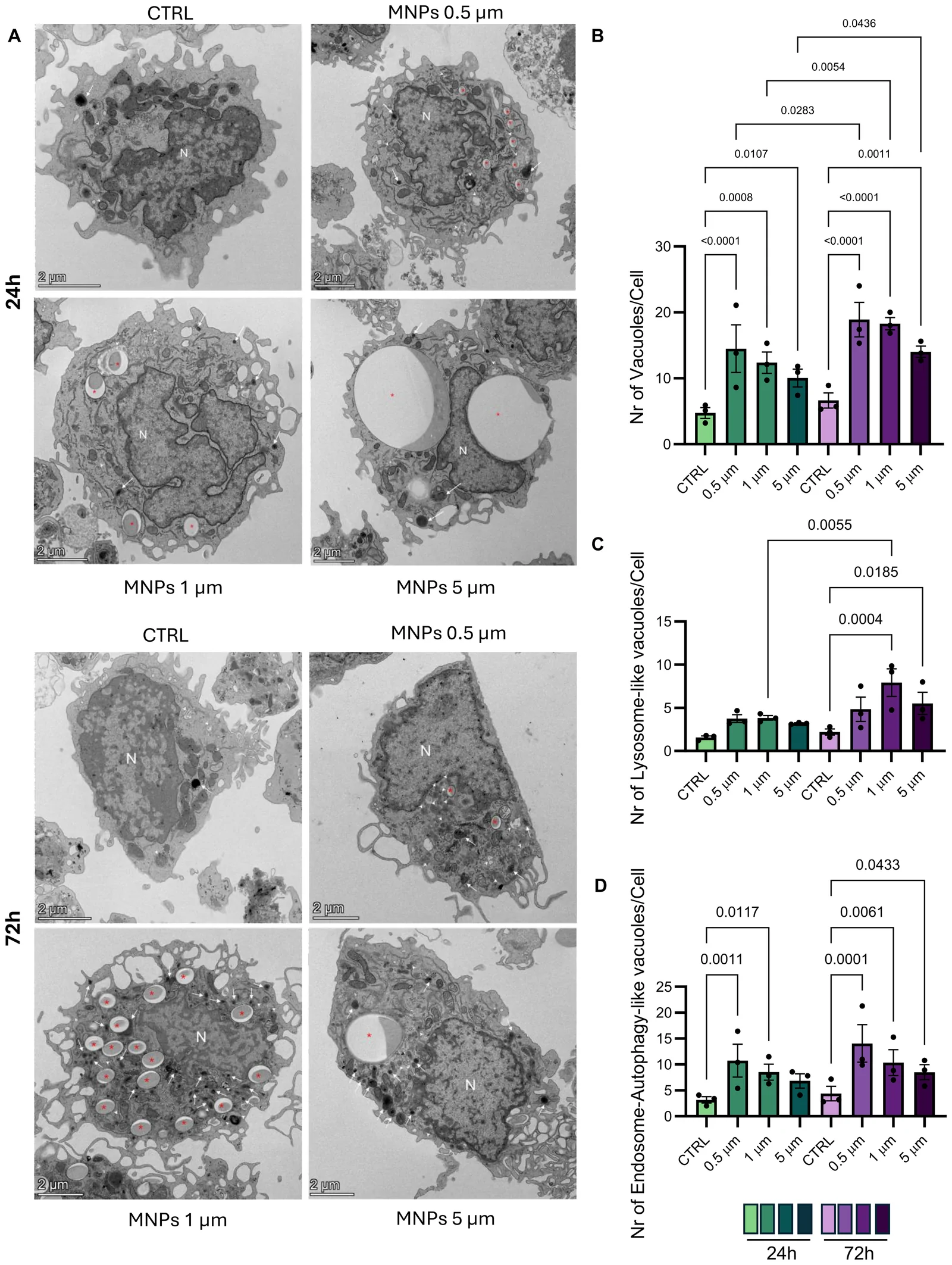
Morphological quantification of intracellular vacuoles. **(A)** Representative TEM images of human monocytes exposed to 25 µg/ml of 0.5, 1, 5 µm MNPs at 24hand 72h and quantification of **(B)** the total number of vacuoles per cell, **(C)** the number of lysosome-like vacuoles per cell, and **(D)** the number of endosome-autophagy-like vacuoles per cell. Results are shown as mean ± SEM. n=3 biological replicates/condition. Two-way ANOVA, followed by Fisher’s LSD *post-hoc* test. White arrows point to lysosome-like vacuoles and arrowheads point to endosome-autophagy-like vacuoles. Red asterisks indicate MNPs; N, nuclei.

Guided by these observations, we next performed morphometric analysis on TEM images (**Figure 4A**) to quantify the total number of vacuoles per cell (**Figure 4B**). Vacuoles were further classified into lysosomes, defined as single-membrane structures with a small, round morphology and a uniformly electron-dense lumen lacking visible cargo (**Figure 4C**), and phagocytic/autophagy-related vacuoles, including (auto)phagosomes, (auto)phagolysosomes, and multilamellar bodies, characterized by single-, double-, or multi-membrane compartments containing cytosolic material, organelles, or partially degraded content (**Figure 4D**).

All MNP sizes increased the number of vacuoles per cell at both time-points, with a clear time-dependent accumulation and a most prominent effect for the smaller particles (**Figure 4B**). The number of lysosomes did not significantly change at 24 h compared with the untreated control, but they accumulated at 72 h across all particle sizes, with the strongest effect observed for 1 µm MNPs, which reached statistical significance compared to 24 h (**Figure 4C**). Finally, phagosome/autophagy-like vacuoles were significantly increased across all the three MNPs sizes, with the most pronounced increase observed for 0.5 µm MNPs and showed no significant differences between 24 h and 72 h (**Figure 4D**). Together, these morphometric data indicate that MNPs, and especially smaller particles, engage and progressively load the autophagy–lysosome system, driving a remarkable lysosomal adaptation/biogenesis over time, while early phagocytic/autophagy-like stages remain comparatively stable across the 24–72 h window.

### 3.3 Biochemical and molecular validation of autophagy-lysosomal engagement by MNPs and their dynamics

**Figure 5:**
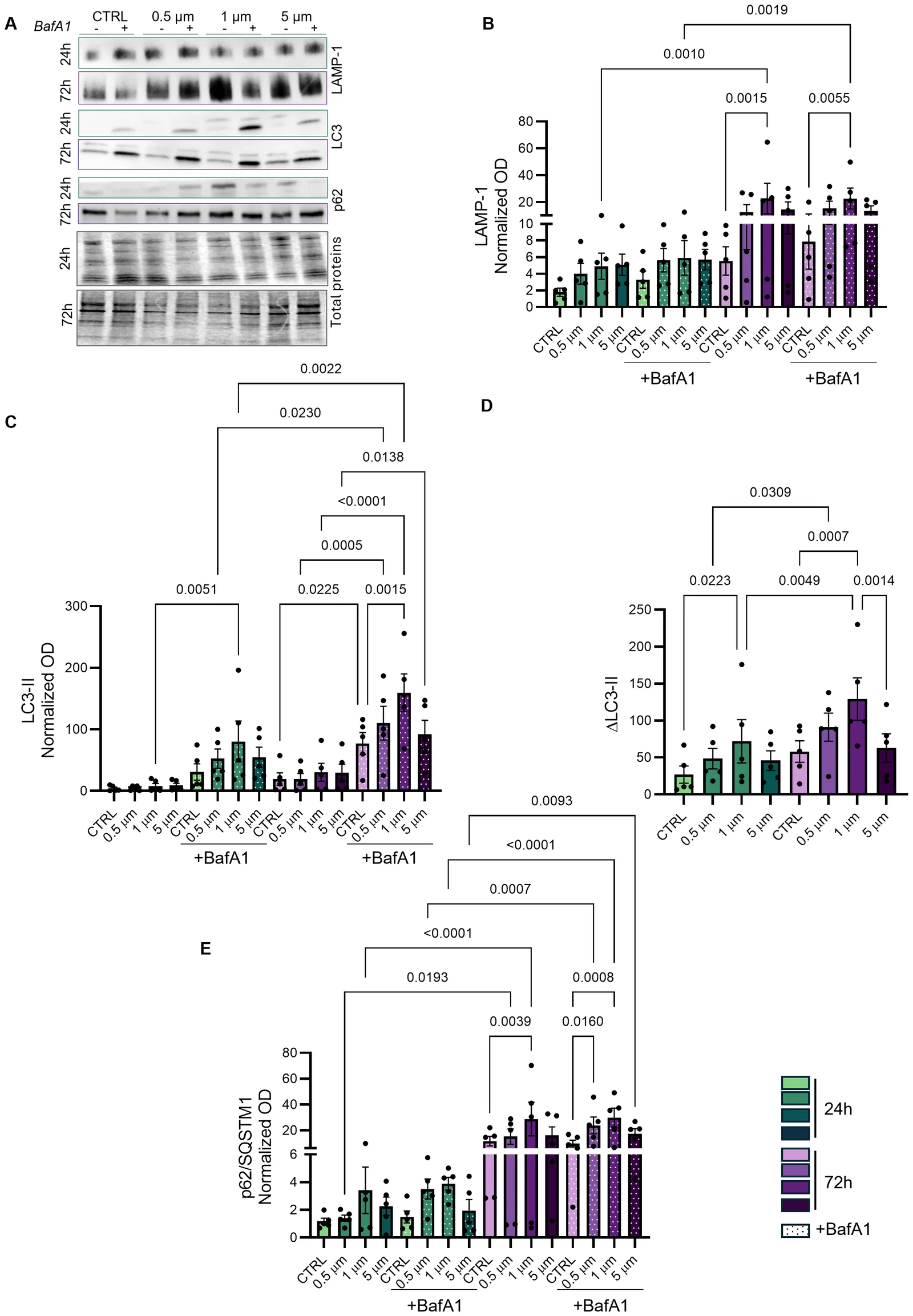
Quantification of autophagy-related markers of human monocytes upon 0.5, 1, 5 µm MNPs exposure. **(A)** Representative Western Blot of LAMP-1, LC3I and LC3II, and p62. Quantification of **(B)** LAMP-1 protein optical density (OD), **(C)** LC3-II OD, **(D)** ΔLC3-II calculated from ODs normalized on total protein of samples treated with Bafilomycin - samples not treated with Bafilomycin, and **(E)** p62 protein (OD) normalized on total protein. Results are shown as mean ± SEM. n=5 biological replicates/condition. Two-way ANOVA, followed by Fisher’s LSD *post-hoc* test.

To evaluate the autophagy-lysosomal axis and its modulation by MNPs, we assessed LAMP-1 (Lysosome-associated membrane protein 1), LC3 (Microtubule-associated protein 1 light chain 3), and p62/SQSTM1 (Sequestosome 1) levels by Western Blot (**Figure 5A**), in the presence or absence of Bafilomycin A1 (BafA1), a V-ATPase inhibitor that prevents lysosomal acidification and autophagosome–lysosome fusion (32).

Monocytes exposed to 0.5, 1, and 5 µm MNPs showed increased LAMP1 protein levels at 72 h post-incubation compared with untreated controls and with cells exposed to the same particle sizes for 24 h (**Figure 5B**), suggesting a time-dependent adaptation and expansion of the lysosomal compartment in response to sustained particle load. As expected, short BafA1 treatment (3 h) did not alter LAMP-1 protein levels (**Figure 5B**). During autophagy, cytosolic LC3 (LC3-I) is lipidated to LC3-II and recruited to autophagosomal membranes, where it accumulates when autophagosome formation increases or lysosomal degradation is impaired (33). As expected, BafA1 treatment induced LC3-II accumulation under all experimental conditions and at both time-points. In the absence of BafA1, LC3-II did not significantly differ between control and MNP-treated cells, suggesting that steady-state LC3-II does not accumulate detectably under these conditions, consistent with a fast-ongoing autophagic turnover. By contrast, for smaller MNP sizes (0.5 – 1 µm), and at both time-points, BafA1-treated monocytes showed significantly higher LC3-II levels compared with untreated controls and the corresponding cells not receiving BafA1 (**Figure 5C**). Consistently, the autophagic flux index, calculated as the difference between BafA1-treated and untreated samples (ΔLC3-II), was higher in small-sized MNP-treated samples than in untreated controls at both 24 and 72 h. In particular, ΔLC3-II was significantly higher in monocytes exposed to 0.5 and 1 µm MNPs and further increased at 72 h compared with 24 h (**Figure 5D**). Together, these data suggest that MNPs exposure enhances biogenesis or accumulation of LC3II-postive vacuoles following MNPs uptake (34), with progressive autophagosome formation overtime, as reflected by ΔLC3-II.

**Figure 6:**
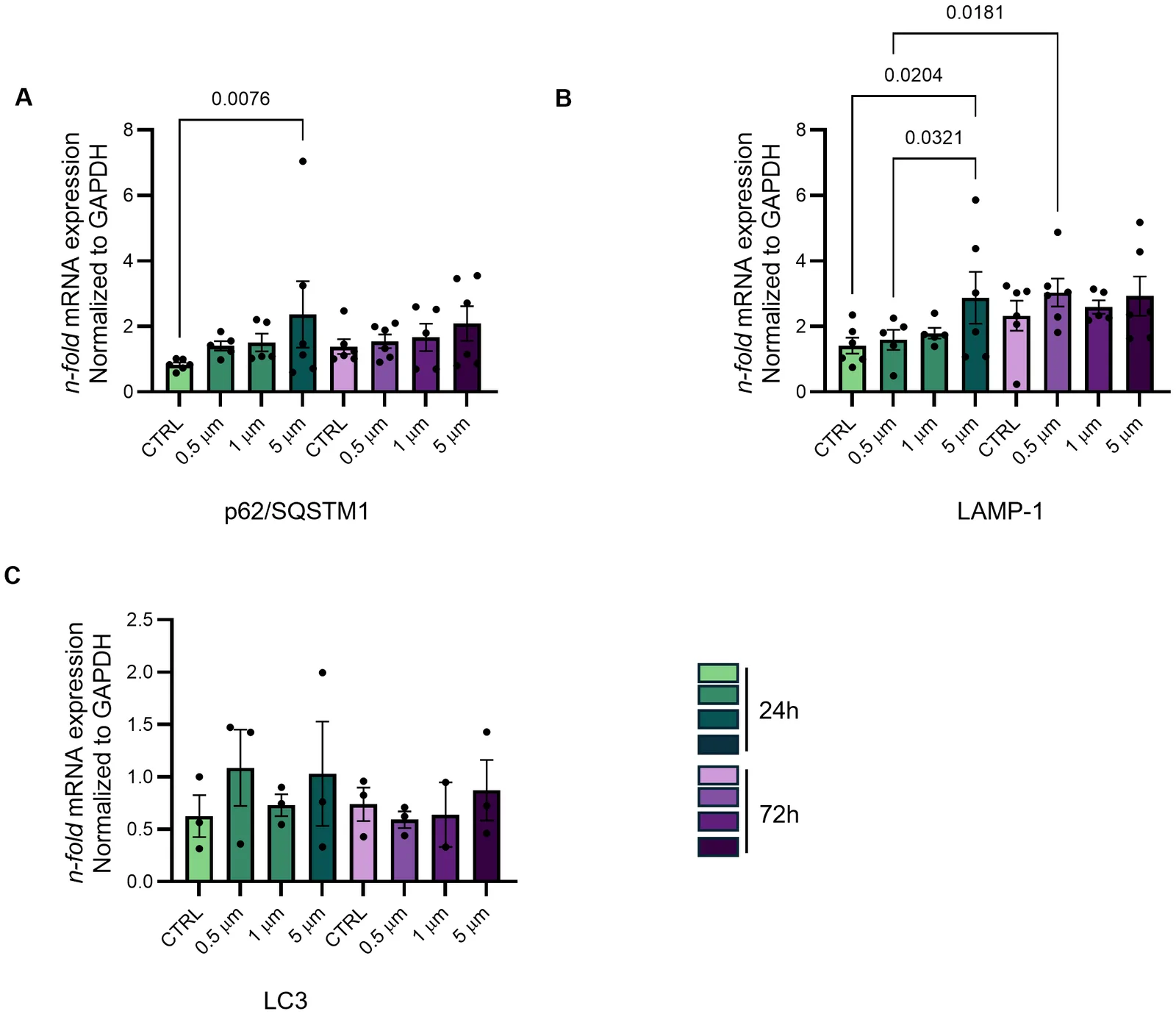
Autophagy-related gene expression in primary human monocytes exposed to MNPs. Real time RT-qPCR analysis of autophagy-related genes, including **(A)** SQSTM1/p62, **(B)** LAMP1, and **(C)** MAP1LC3B/LC3 in control (CTRL) and monocytes exposed to 0.5, 1, 5 μm MNPs (24 and 72 h). Values were normalized to GAPDH and are shown as mean ± SEM. n=3 biological replicates/condition. Two-way ANOVA, followed by Fisher’s LSD *post-hoc* test.

Finally, the autophagy-lysosomal substrate p62/SQSTM1 increased time-dependently across all conditions and it was higher in cells exposed to smaller MNPs, particularly 1 µm particles, compared with untreated controls (**Figure 5E**). At first glance, this pattern could suggest impaired cargo degradation or incomplete autophagic flux under MNPs exposure at later time points. However, p62/SQSTM1 protein levels did not increase upon BafA1 treatment in either controls or MNP-treated cells (**Figure 5E**). This alternatively suggests that p62 abundance may also reflect stress-associated adaptive mechanisms rather than solely impaired degradation (35). In line with this, SQSTM1 mRNA showed early modulation at 24 h, with a significant increase following exposure to 0.5 and 5 µm MNPs (**Figure 6A**). For small MNPs, the delayed increase in p62/SQSTM1 protein levels at 72 h aligns with the earlier transcriptional response observed at 24 h, consistent with a temporally associated stress response. In contrast, although 5 µm particles were associated with a marked increase in SQSTM1 mRNA, this was not accompanied by a corresponding increase in p62 protein levels at 72 h compared with controls, suggesting that p62 degradation may remain active under these conditions (**Figure 6A**). (36).

Consistent with a time-dependent, and stress-related transcriptional remodeling following MNPs exposure, *p62/SQSTM1* mRNA levels correlated with the mRNA expression of genes involved in mitochondrial homeostasis and quality control, including *BAX, NRF1, FIS1, PINK1, OPA1, MTND1, TOMM20*, and *TFAM* (**Supplementary Figure 2** – Spearman correlation). *LAMP1* mRNA levels also increased in response to 0.5 and 5 µm particles (**Figure 6B**), suggesting that lysosomal biogenesis is partially transcriptionally coupled to MNPs exposure. In contrast, *MAP1LC3B* mRNA levels did not change significantly (**Figure 6C**), indicating that the observed autophagosome accumulation occurs predominantly at the post-transcriptional level. The accumulation of autophagosomes (LC3II), and lysosomes (Lysotracker) was further confirmed qualitatively by immunofluorescence staining, where all the MNP sizes showed increased lysotracker signal co-localized with LC3 expression at the later time point (**Supplementary Figure 3**).

### 3.4 Effects of MNPs exposure on mitochondrial metabolism of monocytes

**Figure 7:**
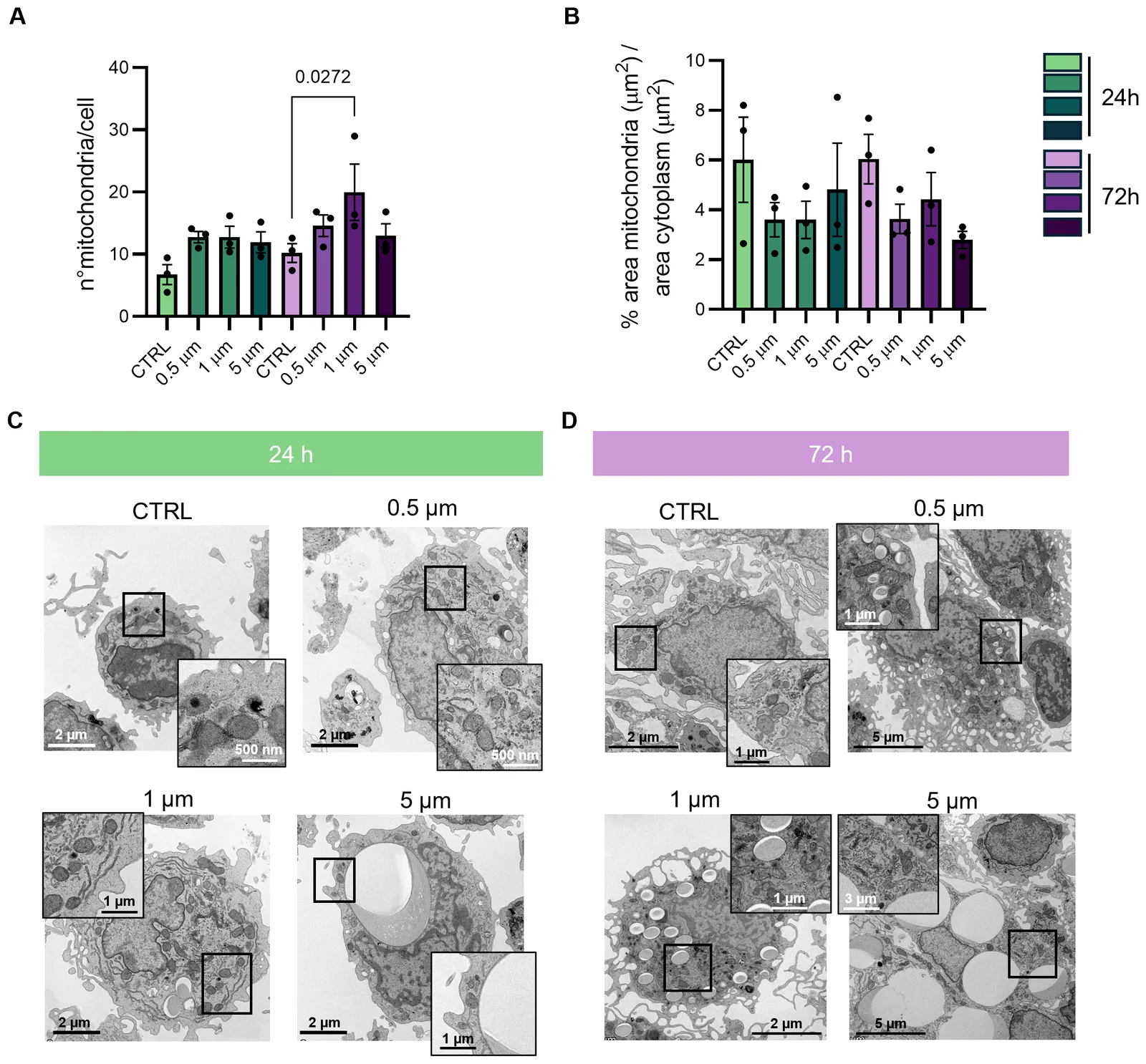
Ultrastructural characterization of mitochondria following MNPs exposure. **(A)** Number of mitochondria per cell. **(B)** Mitochondrial density, calculated as percentage of mitochondrial area relative to cytoplasmic area. Representative TEM images at **(C)** 24 h and **(D)** 72 h. Results are shown as mean ± SEM. n=3 biological replicates/condition. Two-way ANOVA, followed by Fisher’s LSD *post-hoc* test.

Based on the observed internalization of MNPs and their impact on the (auto)phagosome-lysosome axis, we next investigated their effects on cellular metabolic activity. Mitochondrial ultrastructure was first evaluated by TEM imaging. Quantitative analysis revealed an increased trend in the number of mitochondria per cell over time, as well as in MNP-treated compared to untreated cells (**Figures 7A, C, D**). However, this increase was significant only for 1 µm MNPs at 72 h. By contrast, the percentage of mitochondrial area relative to cytoplasmic area showed a trend towards reduction in MNP-treated cells compared to controls (**Figures 7B–D**), suggesting the presence of more numerous though smaller mitochondria upon MNPs exposure. Yet, no significant alterations were observed in mitochondrial circularity or cristae morphology (**Supplementary Figure 4**), indicating preservation of overall mitochondrial ultrastructure.

**Figure 8:**
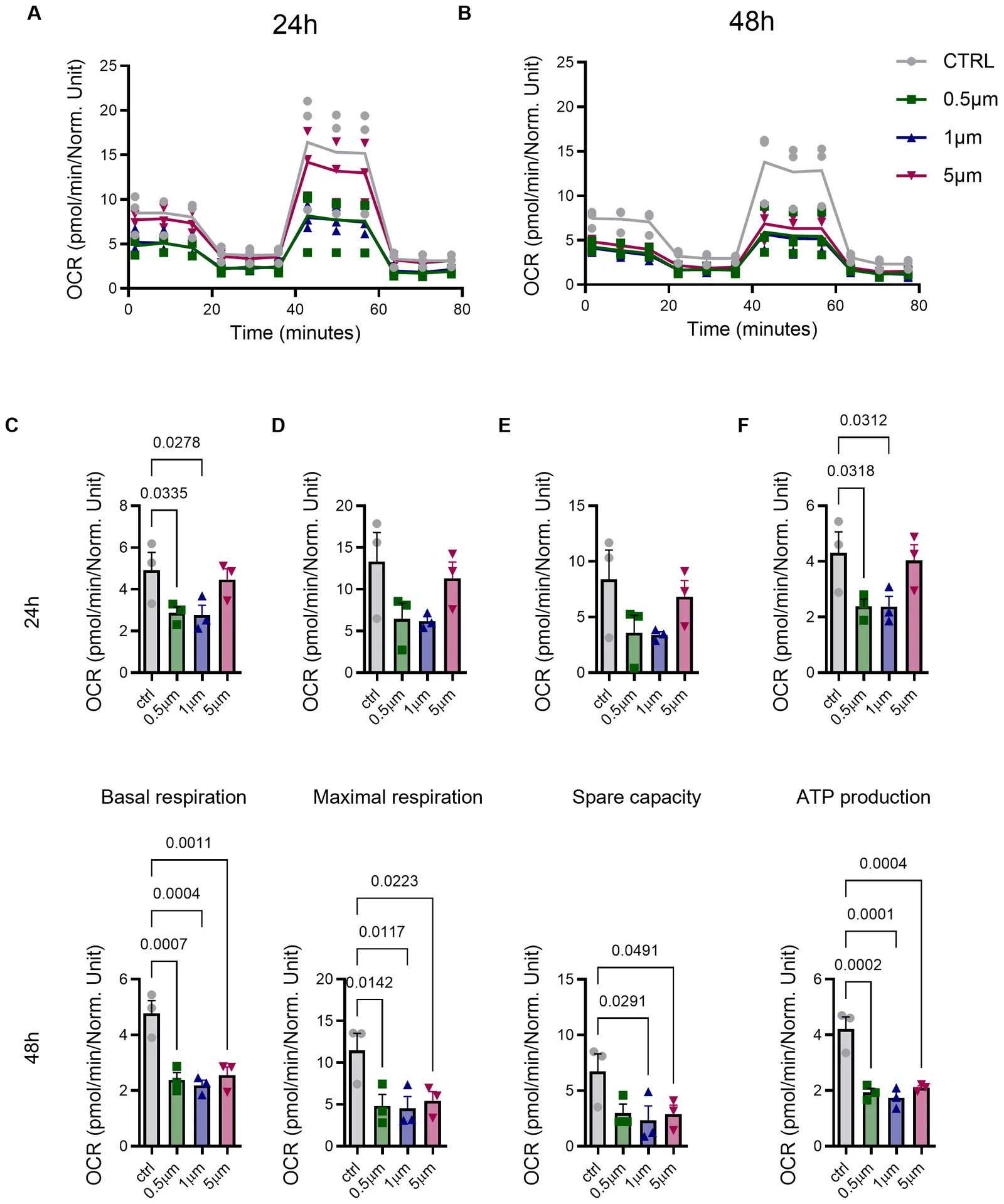
Monocytes mitochondria metabolic activity. Oxygen consumption rate profiles at **(A)** 24 h and **(B)** 48h post exposure to MNPs. **(C–F)** Mitochondrial basal and maximal respiration, spare capacity, and ATP production at 24 h (up) and 48 h (down) post exposure to MNPs. Results are shown as mean ± SEM. n=3 biological replicates/condition. One-way ANOVA followed by Fisher’s LSD *post-hoc* test.

**Figure 9:**
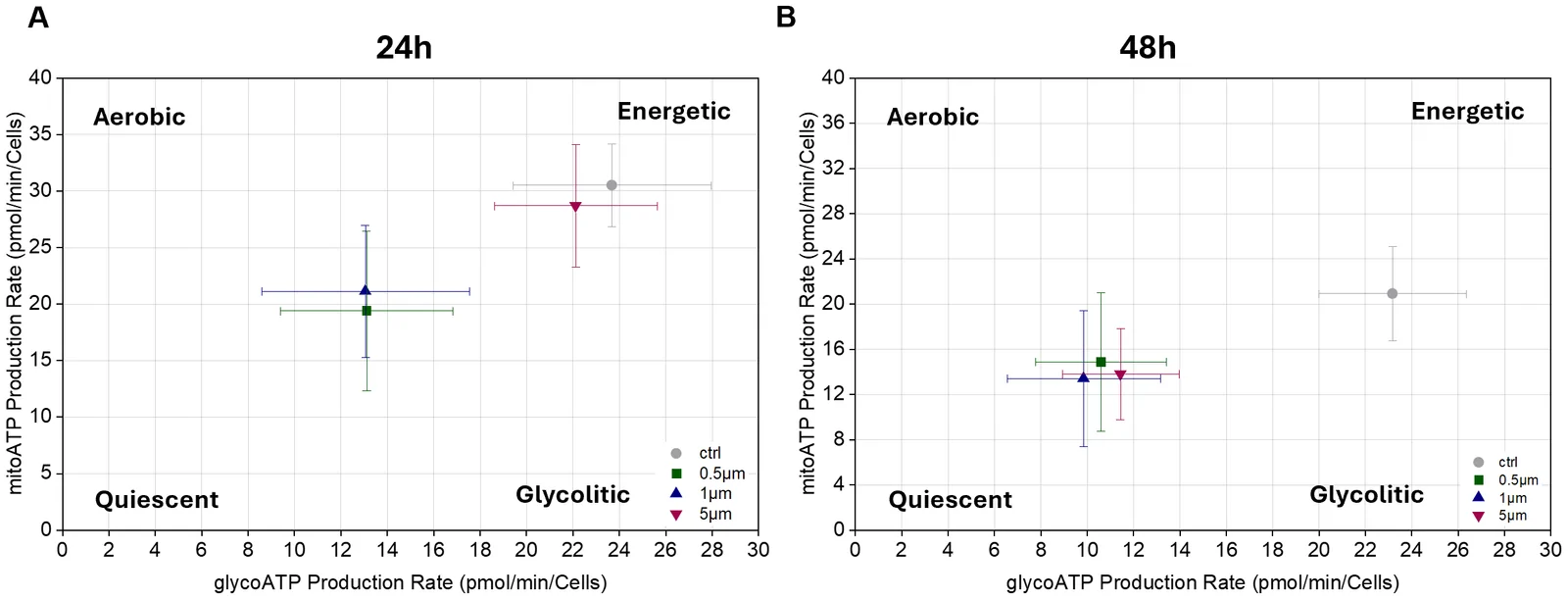
Monocytes energy consumption upon MNPs exposure. Energy map of human-derived monocytes after exposure to 25ug/mL of 0.5, 1, 5um MNPs at **(A)** 24 and **(B)** 48h. Results are shown as mean ± SEM. n=3 biological replicates/condition.

To functionally investigate mitochondrial activity, we analyzed the bioenergetic profile of cells exposed to MNPs for 24h or 48h. Oxygen consumption rate (OCR) profiling revealed distinct profile among the three MNPs sizes (**Figures 8A, B**; **Supplementary Figure 5**). Notably, cells exposed to MNPs showed reduced basal and maximal respiration compared to controls, as well as reduced spare respiratory capacity, which correlate with a decreased ATP produced by mitochondrial respiration (**Figures 8C–F**). The MNP-associated impairment of mitochondrial function appears to be size and time-dependent, as it was rapidly observed (24h) in cells exposed to smaller size MNPs (0.5 µm and 1 µm), while the effect of 5 µm MNPs exposure was less pronounced at 24h but clearly after 48h exposure (**Figures 8A–F**; **Supplementary Figure 5**). Combined analysis of OCR and Extracellular Acidification Rate (ECAR) enabled characterization of the cellular metabolic profile revealing size-dependent alterations in mitochondrial aerobic and glycolytic activity (**Figure 9**). This analysis revealed that monocytes treated with 0.5 µm and 1 µm MNPs are in a quiescent status compared to unexposed controls or monocytes exposed to 5 µm MNPs at the early 24h timepoint. Contrarily, at 48h, monocytes treated with all MNPs sizes exhibited a quiescent status compared to unexposed controls (**Figures 9A, B**). Taken together, these data show an impact of MNPs on cellular metabolic activity, especially for MNPs sizes that are easily internalized into the cell, thus confirming the possible cellular rearrangement.

**Figure 10:**
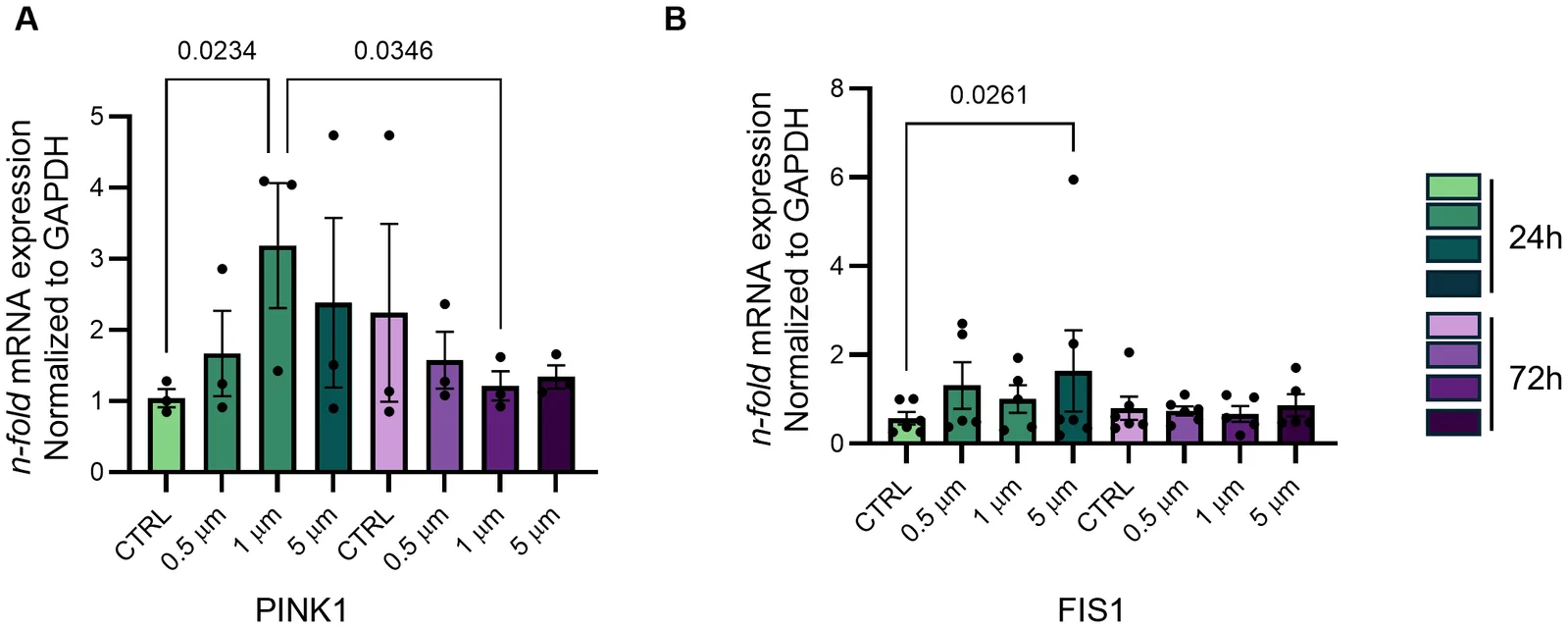
Expression of mitochondria-associated genes in primary human monocytes exposed to MNPs. Real-time RT-qPCR analysis of **(A)** PINK1 and **(B)** FIS1 expression. Results are shown as mean ± SEM. n=3 biological replicates/condition. Two-way ANOVA, followed by Fisher’s LSD *post-hoc* test.

To further evaluate potential mitochondrial rearrangement, we investigated the expression of genes involved in mitochondria biogenesis, dynamics and quality control by qPCR. Among the genes examined, *PINK1* expression was significantly increased at 24 h in cells treated with 1 µm MNPs compared to other conditions (**Figure 10A**), while *FIS1* expression was significantly higher at 24 h in cells treated with 5 µm MNPs (**Figure 10B**), suggesting a possible perturbation of mitophagy-related pathways and fission induction. In contrast, no significant changes were observed in *MT-ND1, TFAM, TOMM20, NRF1* expression suggesting limited transcriptional impact of MNPs on mitochondrial biogenesis and dynamics (**Supplementary Figure 6**).

## 4 Discussion

Micro- and nanoplastics (MNPs) are emerging environmental pollutants widely detected in drinking water, air, food packaging, and household products. Polystyrene (PS) represents one of the major contributors to this exposure burden, owing to its widespread use in consumer goods, including food-contact materials and packaging. Under routine conditions, including food heating, mechanical stress, and environmental degradation, PS-based products can release MNPs, increasing the potential for human exposure to PS-derived MNPs (8, 37, 38). Although MNPs have been detected in human tissues across a wide range of concentrations, the mechanisms linking cellular uptake to intracellular stress responses remain insufficiently understood (8, 10, 29, 30, 39).

Here, we provide an integrated, size-resolved characterization of MNPs internalization and associated ultrastructural intracellular remodeling in primary human monocytes.

Previous studies showed metabolic alterations in MNPs-exposed murine and immortalized human macrophages models. Adler et al. also investigated the effects of MNPs in primary human monocytes focusing on cell viability, reactive oxygen species production, and macrophages polarization (26). However, to our knowledge a systematic comparison of size-dependent effects at the level of ultrastructural internalization, organelles rearrangement and metabolic adaptation on primary human monocytes has not been reported yet.

Importantly, MNP-exposed primary human monocytes exhibited features of functional quiescence, suggesting that particle internalization is associated with substantial changes in intracellular homeostasis. Further studies will be required to determine whether these alterations translate into impaired immune-metabolic function (23, 40–44).

Importantly, a time-dependent viability was observed in both MNP-treated and untreated control monocytes, suggesting increasing baseline stress associated with prolonged culture. As this behavior reflects the limited *ex vivo* stability of unstimulated primary monocytes, all the findings were interpreted relative to time-matched controls.

With high-resolution Transmission Electron Microscopy (TEM) imaging, we resolved the sequential stages of MNPs internalization within primary human monocytes, which documented a series of membrane-associated processes consistent with phagocytic uptake (45). Moreover, MNPs uptake was inversely correlated with their diameter under the tested exposure conditions, suggesting particle-size associated differences in internalization, with 0.5 and 1 µm MNPs being internalized more efficiently than 5 µm particles. However, we should also consider that because all particle sizes were administered with the same mass concentration, the experimental conditions differed for both their diameter and the particle number.

Beyond internalization itself, our findings suggest that MNPs exposure elicits a progressive remodeling of the degradative vacuolar system. The accumulation of multilamellar bodies and hybrid vesicular structures, particularly in response to smaller particles, is consistent with enhanced endo-lysosomal activity and sustained membrane turnover. Morphometric data further support this interpretation, showing expansion of the vacuolar compartment over time and a marked lysosomal increase at 72 h. Together, these findings support a model in which MNPs are efficiently sequestered within the degradative vacuolar system, imposing a progressive burden that is associated with lysosomal biogenesis and remodeling.

Biochemical analyses further supported the ultrastructural evidence of an adaptive autophagy-lysosomal response to MNPs exposure. The early increase in *LAMP1* mRNA followed by later protein accumulation indicates lysosomal induction or expansion in response to 1 µm MNPs. Moreover, LC3-II dynamics and the increased autophagic flux index (ΔLC3-II) indicate a marked expansion of the autophagosomal compartment. This response may reflect activation of canonical autophagy and/or LC3-associated phagocytosis, both of which are consistent with sustained processing of phagocytosed material (34). However, accumulation of LC3-associated vacuoles may also reflect the presence of stagnant autophagy vacuoles resulting from impaired clearance. Analysis of p62/SQSTM1 protein levels increased over time, particularly in response to smaller MNPs (0.5 and 1 µm), but, unlike LC3-II, it did not show further accumulation upon BafA1 treatment. These findings open up two possible scenarios. First, p62/SQSTM1 accumulation may indeed reflect impaired degradative flux following MNPs exposure. In this case, the relatively short BafA1 treatment may have been insufficient to reveal p62 accumulation. Alternatively, p62/SQSTM1 accumulation may arise from its sequestration into degradation-resistant protein aggregates associated with persistent particulate cargo (43), or from increased synthesis driven by stress-responsive adaptive pathways rather than defective degradation itself. In support of this, *SQSTM1* mRNA was induced early, preceding the later increase in protein levels and positively correlated with the expression of mitochondrial genes. Notably, the different pattern observed with 5 µm particles, where strong early mRNA induction was not followed by protein accumulation, suggests that p62/SQSTM1 turnover may remain efficient when particle internalization is limited. Overall, these findings suggest that the accumulation of p62/SQSTM1 is consistent with an adaptive stress response rather than with overt failure of the degradative system (46).

Consistent with previous evidence linking MNPs exposure to cellular stress, our findings highlight mitochondria as key stress-responsive organelles capable of rapidly remodeling their morphology and bioenergetic function under the tested exposure conditions (8, 20, 39, 47–50). Indeed, we show that MNPs exposure induced a trend toward an increased mitochondrial number per cell and reduced mitochondrial area, though ultrastructure was preserved. In parallel, we observed an early modulation of genes involved in mitochondrial dynamics and quality control, including upregulation of PINK1, supporting the activation of mitochondrial surveillance mechanisms in response to MNP-related stress (51, 52). Functional data further indicate that these events are accompanied by impaired mitochondrial performance. In particular, exposure to 0.5 and 1 µm MNPs reduced respiratory capacity already at 24 h, whereas the effect of 5 µm particles was delayed and became detectable only at 48 h. Overall, these findings suggest a size-associated perturbation of mitochondrial homeostasis, with the more pronounced alterations observed for smaller particles potentially related to their higher internalization efficiency and intracellular burden, consistent with previous studies (53, 54). Measurements of OCR and ECAR instead suggests that MNPs exposure drives primary human monocytes toward a quiescent metabolic state. The concomitant reduction in mitochondrial respiration and glycolytic activity is consistent with an overall suppression of bioenergetic metabolism rather than with metabolic activation, contrarily to a previous study employing 10 µm PS-MNPs on murine immortalized macrophage (25).

Overall, our findings support a model in which smaller MNPs (0.5–1 µm) are efficiently internalized by primary human-derived monocytes and are associated with a progressive burden on the autophagy–lysosomal and mitochondrial systems. Rather than indicating a classical lysosomal blockade, this sustained load appears to be accompanied by a transcriptional and post-translational adaptive response characterized by vacuoles remodeling, mitochondrial alterations, and a quiescent metabolic state. In contrast, larger particles (5 µm), which are less efficiently internalized, elicit a more limited intracellular response, identifying particle size as a critical determinant of cellular impact. Under equal-mass exposure conditions, these size-associated effects may be relevant for understanding the biological consequences of MNPs exposure (55, 56).

We acknowledge that the use of polystyrene particles under controlled experimental conditions does not fully capture the physicochemical heterogeneity of environmental MNPs, which differ in composition, shape, and surface properties. Furthermore, although the selected concentrations produced robust and reproducible responses, they may not directly reflect environmental exposure levels. Moreover, future studies using complementary approaches such as tandem fluorescent LC3 reporters, extended BafA1 time-course experiments or p62 solubility analyses will be seminal to provide a more definitive assessment of autophagic flux upon MNPs exposure. Likewise, assessing protein levels of mitochondrial mediators which we documented at the transcriptional level such as PINK1 and FIS1, will be critical to provide additional information on mitochondrial quality-control pathways. Moreover, it is important to acknowledge that the primary human monocytes used in this study were obtained from anonymized healthy donors. This may have contributed to inter-donor variability and should be considered when interpreting our findings.

Despite these limitations, our study suggest that smaller plastic particles may exert biologically relevant effects on innate immune cell homeostasis even under 25 µg/mL exposure conditions. This may be particularly relevant for macrophages residing in highly specialized microenvironments, including lung, gut, liver, brain, placenta, and decidua, where impaired myeloid cell function could affect local immune regulation and tissue integrity.

Collectively, our findings provide a foundation for future studies aimed at determining the functional consequences of MNPs exposure in myeloid cells and clarifying how these alterations may translate into pathological outcomes and long-term health effects.

## Data Availability

The raw data supporting the conclusions of this article will be made available by the authors, without undue reservation.
