# Supplementary material for: Micro- and nanoplastics remodel the autophagy-lysosomal axis and mitochondrial function in primary human monocytes
Source: Front Immunol. 2026 Aug 26;17:1919477. doi: 10.3389/fimmu.2026.1919477 (PMC13553408; doi:10.3389/fimmu.2026.1919477)
Supplement: Supplementary file 1 [file SupplementaryFile1.docx]

Supplementary Material

# Supplementary Figures

**Supplementary Figure 1. Flow cytometry gating strategy for THP-1 cell line.** Representative gating strategy used to identify live and dead THP-1 cells upon exposure to MNPs of different concentrations and diameter, and to distinguish MNP-negative from MNP-positive (MNP+) cell populations.

**Supplementary Figure 2.** **Exploratory Spearman correlation analysis performed using paired mRNA and protein data about autophagy and mitochondrial-related markers (n=24 paired observations from n=3 independent donors).** (**A**) Spearman correlation coefficient and (**B**) statistically significant (< 0.05) p-values.

**Supplementary Figure 3. Representative immunofluorescence images of lysosomes at 72h post exposure to MNPs.** Nuclei were stained with DAPI (blue), LC3 was detected in green, and lysosomes were labeled with LysoTracker (red).

**Supplementary Figure 4. Ultrastructural mitochondria analysis.** Mitochondrial **(A-B)** area, **(C-D)** diameter, **(E-F)** circularity index measured from TEM images. Results are shown as mean ± SEM. n=3 biological replicates/condition. One-way ANOVA.

**Supplementary Figure 5. MitoStress single donor OCR profiles** of (**A**) Donor1, (**B**) Donor 2, (**C**) Donor3 at 24h and 48h post exposure to 0.5, 1, and 5µm PS-MNPs or untreated control. Data are shown as mean ± SEM of n=6-8 technical replicates.

**Supplementary Figure 6. Expression of genes involved in mitochondrial homeostasis.** Real time RT-qPCR analysis of (**A**) *MT-ND1* (**B**) *TFAM* (**C**) *TOMM20* (**D**) *NRF1* in untreated control primary human monocytes (CTRL) and monocytes exposed to 0.5, 1, 5 MNPs for 24 and 72 h. Gene-expression levels were normalized to GAPDH and are shown as mean ± SEM. n=3 biological replicates/condition. Two-way ANOVA, followed by Fisher’s LSD post-hoc test.

**Supplementary table**

**Supplementary Table 1. Summary of PS-MNPs characteristics.** Diameters (μm) of fluorescent and non-fluorescent PS-MNPs and the corresponding concentrations, expressed as %weight/volume (%w/v) and number of spheres/mL.
